# Surgical management outcome of cerebral schistosomiasis: a case report and review of the literature

**DOI:** 10.1186/s13256-021-02828-z

**Published:** 2021-05-23

**Authors:** Moayad Moawia ZainElabdin Ahmed, Haytham Hussein Mohammed Osman, Alaa Hatim Ameer Mohamed, Alaaeldin Ginawi

**Affiliations:** 1 Department of Neurosurgery, Aliaa specialist hospital, P.O.Box 2613, 11111 Omdurman, Khartoum, Sudan; 2 The National Ribat University, Neurospine Center, Ribat University Hospital, Khartoum, Sudan; 3 Histo-center, Khartoum Hospital Street, Khartoum, Sudan; 4 Queens Medical Centre, Nottingham University Hospitals, Nottingham, UK

**Keywords:** Cerebral schistosomiasis, Magnetic resonance imaging, Antiparasitic treatment

## Abstract

**Background:**

Schistosomiasis is a parasitic infection that commonly affects the gastrointestinal and genitourinary tracts. Cerebral schistosomiasis is rare, and few operative cases have been reported in the literature. Diagnosis is usually challenging due to the similarity of the lesion to many other brain conditions. Treatment usually requires surgical resection combined with the use of antiparasitic agents, which often results in good outcomes and excellent prognosis.

**Case presentation:**

A 24-year-old, previously healthy Afro-asiatic man presented to our neurosurgical outpatient clinic complaining of headache and an attack of convulsions. On examination, he had bilateral lower limb weakness more on the right side. Laboratory investigations including stool and urine general test results were unremarkable. Magnetic resonance imaging of the brain was performed and showed an intra-axial left parietal mass; a granulomatous lesion was suggested in the differential diagnoses. The patient underwent craniotomy and total resection of the lesion. Histopathology confirmed the presence of active cerebral *Schistosoma mansoni* infection. Orally administered praziquantel was initiated at a dose of 20 mg/kg twice a day for a total of 3 days along with oral administration of corticosteroids for 2 weeks. The patient improved postoperatively without residual weakness and with no further convulsions.

**Conclusion:**

Cerebral schistosomiasis is a rare but important consideration in the list of differential diagnoses of cerebral space-occupying lesions. This is of particular importance in in endemic areas like Sudan. In order to reach a diagnosis, careful social and occupational history need to be obtained and correlated with the clinical, laboratory, and radiological findings. Surgical resection along with the use of proper antiparasitic agents usually provides the best clinical outcomes.

## Background

Schistosomiasis is estimated to affect more than 200 million people worldwide, who are infected by contact with contaminated water [1]. Central nervous system manifestations are a result of the inflammatory response to egg deposition; recent infection is usually not present [2]. The commonest cause of cerebral schistosomiasis is *Schistosoma japonicum* [3], but there are many cases due to *Schistosoma mansoni* reported in the literature [4]. We report a rare case of active cerebral schistosomiasis in a young male patient presenting with headache and convulsions, where a lesion occupying the left parieto-occipital space was revealed on magnetic resonance imaging (MRI). The pathology, clinical presentation, diagnostic evaluation, and methods of pre- and postoperative treatment of cerebral schistosomiasis are reviewed.

## Case presentation

**Fig. 1:**
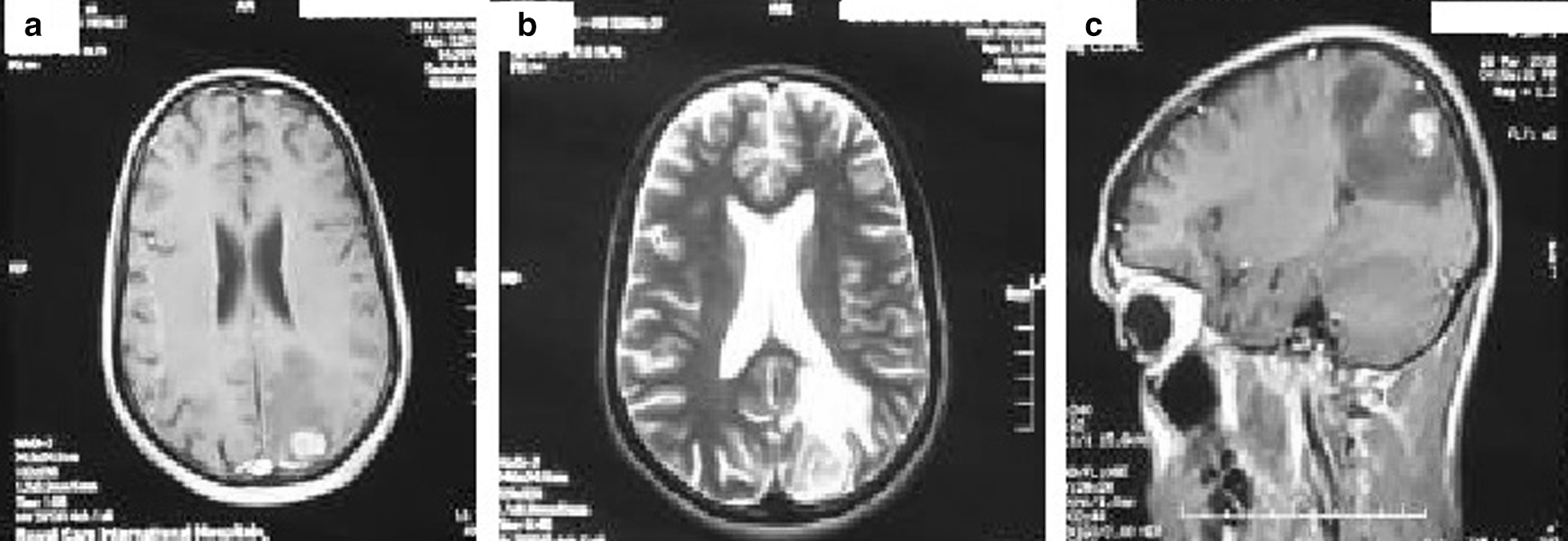
MRI brain showed left posterior parietal homogenous 1.8x1cm enhancing lesion in **a**) axial T1WI with peri-focal oedema. Lesion fairly defined, irregular, lobulated, intra-axial, cortical based rather than dural based, hyper-intense in **b**) axial T2WI and moderate size peri-lesional oedema in **c**) sagittal T1WI with contrast

A 24-year-old, previously healthy Afro-asiatic man presented to our neurosurgical outpatient clinic complaining of headache for 3 months, which was followed by one attack of generalized tonic–clonic convulsions, which prompted him to seek medical advice. He reported no other associated signs or symptoms. Upon general examination, the patient looked unwell, but no pallor, jaundice, or cyanosis was observed. His vital signs were all within the normal range. On neurological examination, he was conscious, oriented to time, place, and person, with a Glasgow Coma Scale score of 15 out of 15 and normal papillary reaction and size bilaterally. The patient had power grade 4+ in both the upper and lower right limbs, with normal tone and reflexes in all joints. Systemic review was unremarkable. Laboratory investigations including stool and urine general test results were unremarkable, so no further cerebral spinal fluid analysis was pursued. Initial plain head computed tomography (CT) showed a focal area of high density within the left parietal region. An MRI of the brain was then performed and showed a fairly well-defined 1.8 × 1 cm lobulated, cortical-based intra-axial lesion within the left posterior parietal region. The lesion demonstrated homogeneous enhancement and had associated moderate perilesional vasogenic edema with only a mild localized mass effect. No other focal lesions were seen (Fig. 1). A list of possible differential diagnoses was made and included granulomatous lesion, lymphoma, a primary glial neoplasm, and less likely meningioma.

**Fig. 2:**
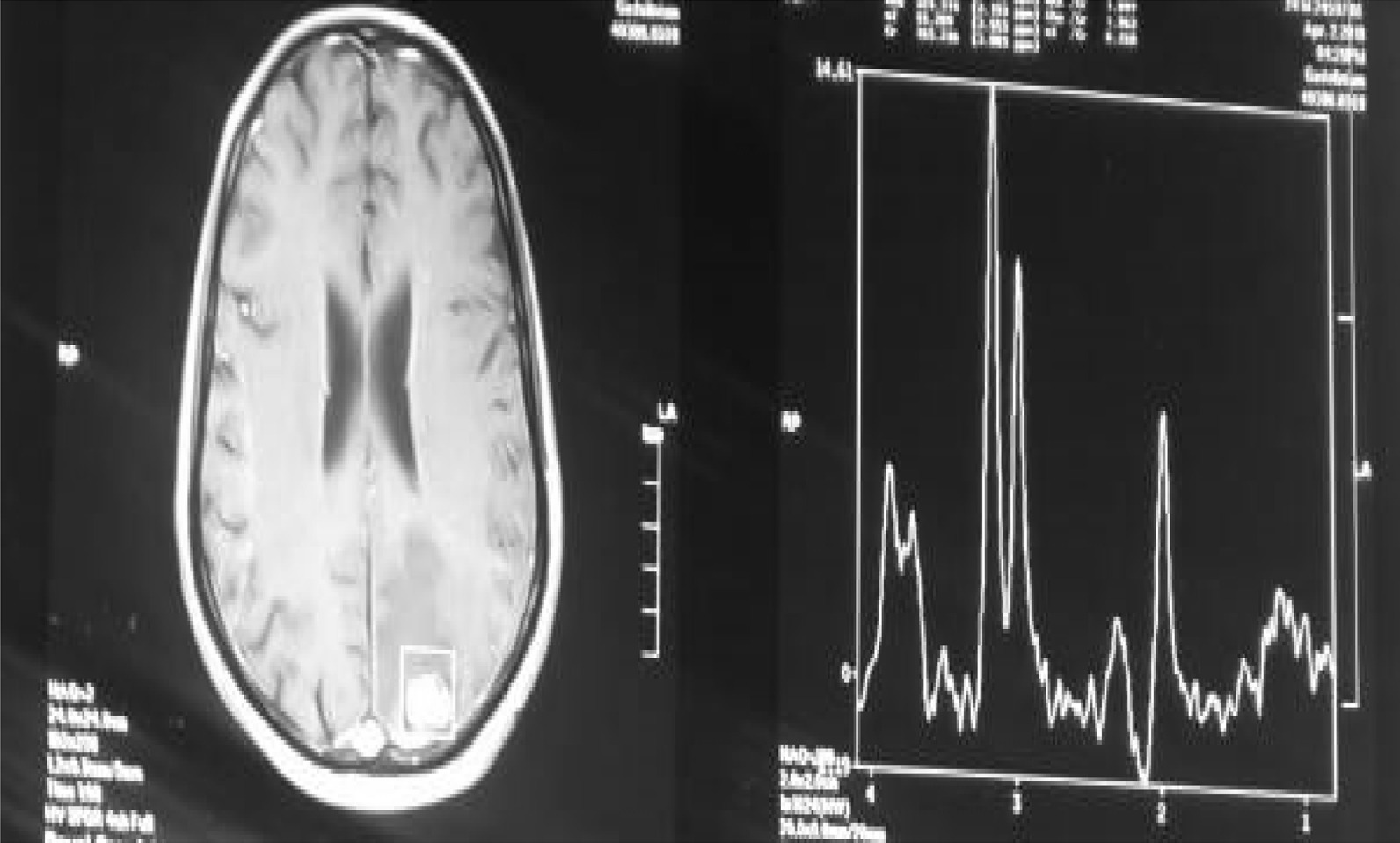
Magnetic resonance spectroscopy showing choline peak suggesting a process causing rapid cell membrane turnover

**Fig. 3:**
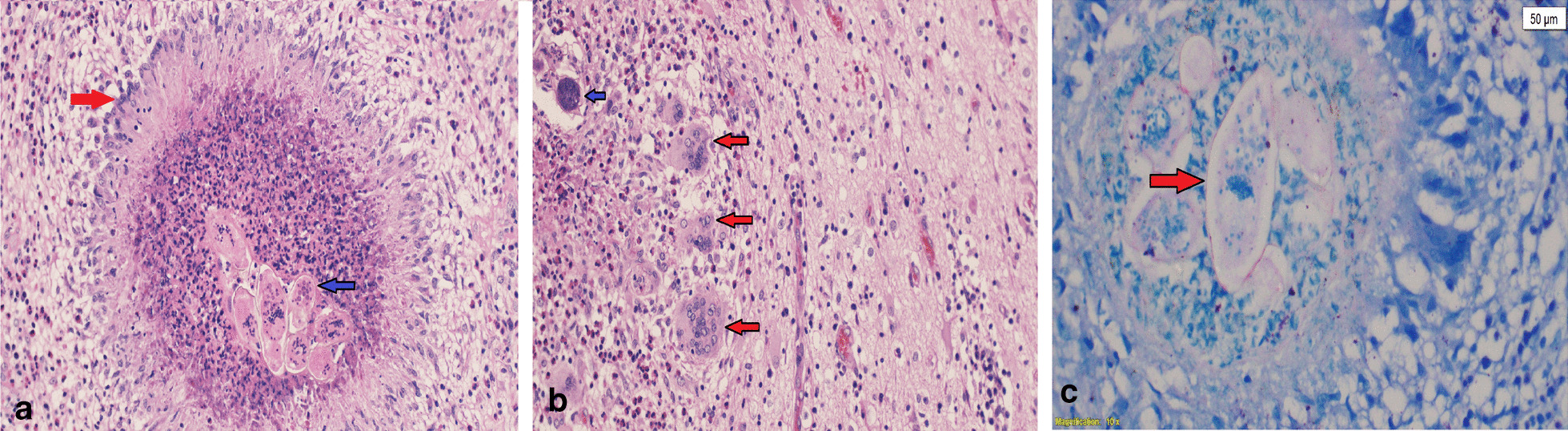
**a**–**c**: Histological sections from hematoxylin and eosin and Ziehl–Neelsen-stained slides, respectively, showing multiple viable *Schistosoma* eggs (blue arrows in **a**, **b**) surrounded by palisading histiocytes (red arrow in **a**) forming necrotizing granuloma associated with mixed inflammatory cell infiltrates composed mainly of eosinophils (**a**, **b**) and giant cell reaction (red arrow in **b**) (magnification ×10 and ×40, respectively). Ziehl–Neelsen-stained slide shows faint positive stain in the shell of a *Schistosoma* egg (red arrow in **c**) consistent with the *Schistosoma mansoni* type (magnification ×10)

**Fig. 4:**
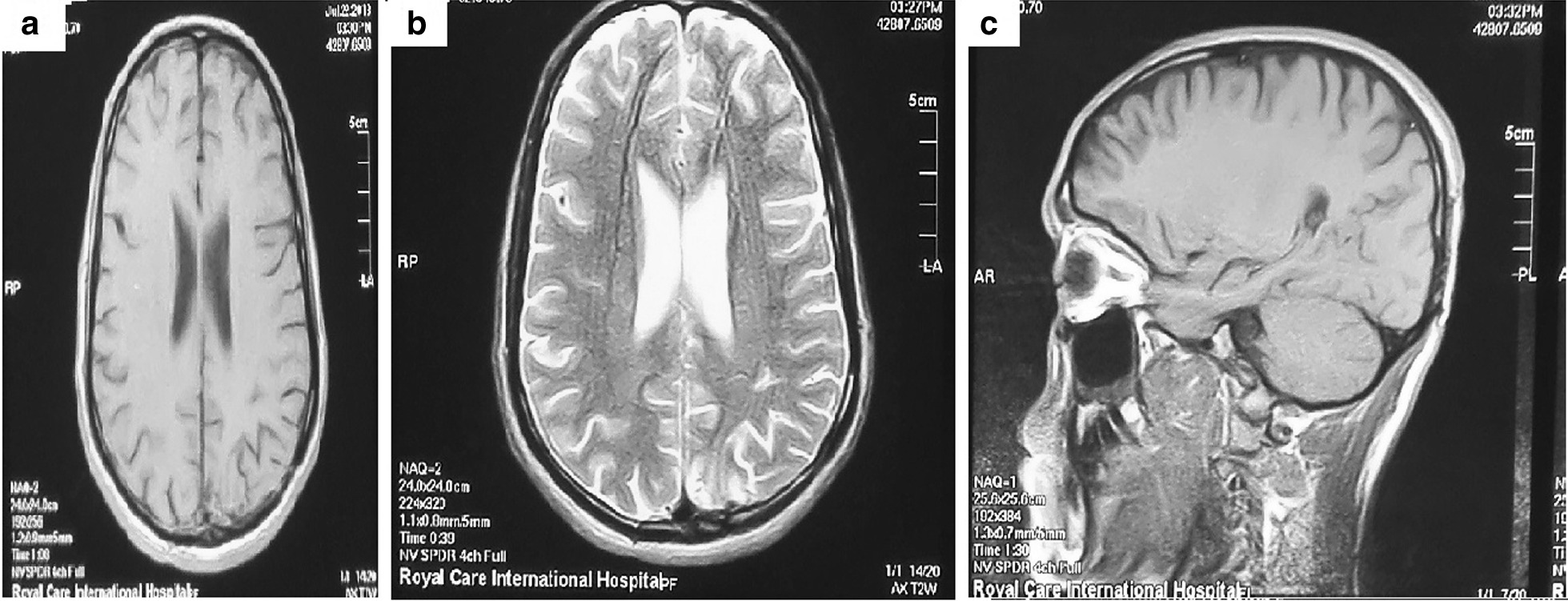
Post-operative MRI brain showed complete resection of the lesion in **a**) axial T1WI, **b**) axial T2WI showed left posterior parietal area of gliosis, **c**) Sagittal T1WI

Magnetic resonance spectroscopy, although nonspecific, demonstrated a choline peak suggestive of cell membrane turnover that can be seen in a number of different conditions including neoplasms, demyelination, inflammation, and gliosis. Correlating this with the patient age and history, an inflammatory lesion was thought to be more likely (Fig. 2). The patient was then admitted and underwent craniotomy and total microsurgical resection of the lesion. Histopathology results showed viable and calcified *Schistosoma mansoni* ova with marked mixed inflammatory infiltrate composed of granulomas and eosinophils, all features consistent with active cerebral schistosomiasis (Fig. 3). Oral administration of antiparasitic praziquantel was initiated at a dose of 20 mg/kg twice a day for a total of 3 days along with orally administered corticosteroids for 2 weeks. The patient was discharged in good condition with improving neurological condition. A postoperative follow-up MRI was performed 3 months later and showed complete resection of the lesion (Fig. 4). Marked neurological improvement was noted at 1-month, 3-month, and 6-month follow-up visits. The patient regained full power and had no further convulsions.

## Discussion

It is estimated that more than 200 million people worldwide are infected with schistosomiasis after contact with contaminants [1]. Five species are known to infect humans, with *Schistosoma japonicum* the most common species affecting the human nervous system [3]. Contact with water that carries the larva of the parasite may result in schistosomiasis [5]. After direct contact, the larva enters the human blood through the skin. Days later, worms migrate to the inferior mesenteric vessels, and the production of eggs begins within 6 weeks following infection [5]. This process continues for the whole life of the worm, or about 3–5 years [6]. The eggs pass through the blood vessel lumen and the intestinal mucosa; they terminate in fecal materials, and the life cycle ends when the eggs release miracidia [5]. The cerebral form of schistosomiasis is thought to result from migration of the worms through the vertebral venous plexus (Batson plexus); in this valveless system, worms are able to migrate and produce eggs directly in the brain [6].

Schistosomiasis of the central nervous system occurs very rarely, with *Schistosoma japonicum* commonly causing cerebral lesions, and *Schistosoma mansoni* and *Schistosoma haematobium* commonly causing spinal cord lesions. *Schistosoma mansoni* and *Schistosoma haematobium* are unusual causes of cerebral mass lesions [7]. Pittella *et al.* reported four patients with cerebral *Schistosoma mansoni* infection and reviewed a few cases that were reported then in the literature [8].

Central nervous system manifestations are a result of the inflammatory response to egg deposition in the brain and spinal cord, and are usually seen in patients with recent infection with no evidence of systemic illness [2]. Supra- or infratentorial foci may result in headache, seizure, and other signs of raised intracranial pressure. Cerebral schistosomiasis is more common in *Schistosoma japonicum*, but an increasing number of cases due to *Schistosoma mansoni* have been reported in the literature [4, 6].

Schistosomiasis of the spinal cord is a rare condition but is well reported in the literature, especially in sub-Saharan African countries, where several case reports and series have been published. Spinal cord schistosomiasis may present clinically by myelopathy, radiculopathy, or both [9].

In Sudan, a case series was reported in which 5 of 200 patients with spinal cord compression were found to have schistosomiasis and underwent surgery and medical treatment, with excellent outcome; diagnosis of those who underwent surgery was confirmed only after histopathological examination [10]. Another case series of 27 patients was reported by El Malik *et al.*, where all patients underwent regular blood tests, serological investigations, and radiological examination, among which two underwent surgical intervention due to expansion of the lesion with no response to medical management [11].

Operative cases of cerebral schistosomiasis are very unusual and have been reported in the literature on rare occasions. *Schistosoma japonicum* eggs in the brain were first described by Tsunoda and Shimamura in 1906 [12]. In 1936, Egan *et a*l. reported two neurological cases of *Schistosoma japonicum* among 12 English sailors who went swimming in the Yangtze River [13].

Patients have been incorrectly diagnosed as having brain tumors, specifically gliomas, due to radiological findings consistent with glioma on CT and MRI [2]. Heterogeneous enhancement was seen mainly at the cerebellum and occasionally at the thalamus, parietal, occipital, and frontal regions [6, 8].

Sanelli *et al.* reported what they called an arborized enhancement pattern with central linear enhancement, which may be significant for cerebral schistosomiasis [14]. Huang *et al.* concluded that diffusion-weighted MRI with apparent diffusion coefficient values may be a useful tool in the diagnosis of cerebral schistosomiasis [15]. A combination of laboratory and radiological investigations are required in order to reach diagnosis in certain cases [2].

Diagnosis of cerebral *Schistosoma* infection is a challenging task. Kato-Katz thick-smear stool examination, which is the most practical laboratory examination for the investigation, can determine the presence of eggs in feces [16]. Imai* et al.* reported the first case of cerebral schistosomiasis due to *Schistosoma haematobium*, which was diagnosed by molecular methods; polymerase chain reaction assay is a promising method for definitive diagnosis and species identification in cases of cerebral schistosomiasis when *Schistosoma* ova in urine or stool specimens can be identified [17]. Histopathological examination remains in the only method for reaching a definite diagnosis [1].

The therapeutic strategy in patients with new-onset seizures should be based on the type of seizures and on the epilepsy syndrome. Long-term antiepileptic drug treatment is typically not indicated [6]. The treatment of cerebral schistosomiasis is highly effective and safe; total or partial resection of the lesion is needed to confirm diagnosis and to relieve the signs and symptoms [2]. Medical treatment after diagnosis with oxamniquine and praziquantel, which are the most effective antiparasitic drugs for treating schistosomiasis, is essential [6] to kill the adult worms, and corticosteroids reduce granulomatous inflammation and are used for all *Schistosoma* subtypes [18].

## Conclusion

Surgical management of cerebral schistosomiasis is safe and highly effective in cases where the diagnostic laboratory and radiological findings are inconclusive, leading to difficulty in initiating appropriate medical management. Surgically, total resection of the lesion is the best choice for confirming diagnosis and alleviating symptoms. Medical treatment should be started after surgery and confirmed diagnosis; oxamniquine and praziquantel are effective antiparasitic drugs. These drugs, used alongside corticosteroids, kill the adult worms and reduce the granulomatous inflammation, respectively.

## Data Availability

All data generated or analyzed during this study are included in this published article and its supplementary information files.

## Abbreviations

MRI: Magnetic resonance imaging
CT: Computed tomography
